# USP13 functions as a tumor suppressor by blocking the NF-kB-mediated PTEN downregulation in human bladder cancer

**DOI:** 10.1186/s13046-019-1262-4

**Published:** 2019-06-14

**Authors:** Xiaojun Man, Chiyuan Piao, Xuyong Lin, Chuize Kong, Xiaolu Cui, Yuanjun Jiang

**Affiliations:** 1 Department of Urology, First hospital of China Medical University, No.155 Nanjing north Road, Shenyang, 110001 Liaoning China; 2 Department of Pathology, The First Affiliated Hospital and College of Basic Medical Sciences, China Medical University, Shenyang, 110001 China

**Keywords:** USP13, PTEN, NF-kB, Bladder cancer

## Abstract

**Background:**

USP13 has been reported to be involved in the tumorigenesis of human cancers, however, its functional role and regulatory mechanisms in bladder cancer (BC) remain unclear.

**Methods:**

q-RT-PCR was performed to examine the expression of miR-130b-3p, miR-301b-3p and USP13 in BC tissue samples. Western blot, q-RT-PCR, bioinformatic analysis and dual-luciferase reporter assay were conducted to identify the regulatory function of miR-130b-3p/301b-3p for USP13. Co-immunoprecipitation assay was performed to assess the interaction between USP13 and PTEN protein. Cell-counting-kit 8, colony formation assay and transwell assay were performed to value the proliferative, migrative and invasive capacities of BC cells in vitro. Mouse xenograft model of BC cells was established to verify the function of USP13 in vivo. Immunohistochemistry was performed to identify the protein expression of USP13, NF-kB p65 or PTEN in clinical/xenograft tumor tissues.

**Results:**

Our present study reveals that USP13 functions as a tumor suppressor by interacting with PTEN protein and increasing its expression in bladder cancer. We found that loss of USP13 led to the downregulation of PTEN and promoted proliferative, invasive and migrative capacities of bladder cancer cells. Furthermore, we discovered that USP13 was a common target of miR-130b-3p and miR-301b-3p, and the miR-130b/301b cluster, which could be transcriptionally upregulated by NF-kB. Our data demonstrated that NF-kB activation decreased expression level of USP13 and PTEN, and promoted the tumorigenesis phenotypes of BC cells. In addition, reintroduction of USP13 partially rescued PTEN expression as well as the oncogenesis trend caused by NF-kB.

**Conclusion:**

We reported a potential regulatory loop that the NF-kB-induced miR-130b/301b overexpression decreased USP13 expression and subsequently resulted in the downregulation of PTEN protein and promoted tumorigenesis of bladder cancer. Moreover, NF-kB-mediated PTEN downregulation is very likely to facilitate the full activation of NF-kB.

**Electronic supplementary material:**

The online version of this article (10.1186/s13046-019-1262-4) contains supplementary material, which is available to authorized users.

## Background

Bladder cancer (BC) is the 10th most common form of cancers worldwide, with an estimated 549,000 new cases and 200,000 deaths in 2018 [1, 2]. In man, it is the sixth most common cancer and ninth leading cause of cancer death [1–3]. Among these tumors, 95% are transitional cell (urothelial) carcinoma (TCC) [4, 5]. There are two variants of TCC: non-invasive and invasive tumors. 80% of the newly diagnosed BC patients are non-invasive carcinoma, however, nearly 15% of these patients will suffer tumor progression and eventually develop a high-risk muscle-invasive BC, which is a lethal disease [4, 5]. Despite the application of programmed cell death protein (PD-1) and programmed death ligand 1 (PD-L1) immune checkpoint therapies has launched a new era for BC treatment [6–8], the study of critical biomarkers and molecular mechanisms for progression of bladder cancer is still imperative.

The phosphatase and tensin homologue deleted on chromosome 10 (PTEN) catalyzes the conversion of phosphatidylinositol-3,4,5-trisphosphate to phosphatidylinositol-4,5-bisphosphate, therefore antagonizes PI3K-Akt signaling [7, 9]. PTEN is located at 10q23, which is one of the most frequently mutated genes in human cancers [10, 11]. PTEN deficiency has been identified in a PTEN-deficient mice model. It contributes to initiation and progression of bladder cancer both by initiating superficial papillary TCC and by promoting the progression of carcinoma in situ (CIS) to advanced tumors [12]. Besides, PTEN protein expression is observed to be reduced in advanced bladder cancer and correlated with cancer stage and grade [13–15]. Despite the role of PTEN in cancers has been firmly established and PTEN gene is frequently altered in human tumors, it is noteworthy that only 25% of cancer patients reveal a correlation between loss of PTEN protein and loss of PTEN mRNA [16]. This emphasizes the importance of post-transcriptional and post-translational regulation for PTEN in PTEN-deficiency-mediated tumorigenesis. Post-translational modification such as phosphorylation, acetylation and ubiquitylation regulates expression, localization and stabilization of PTEN protein [17, 18]. PTEN can be targeted by ubiquitin ligases for proteasomal degradation, on the other hand, PTEN-ubiquitylation can be antagonized by deubiquitylating enzymes (DUBs) [19–21]. Ubiquitin-Specific Protease 7 (USP7, also known as HAUSP, the herpes virus-associated USP) has been implicated in deubiquitylation of PTEN as well as other tumor suppressors such as p53 and FOXO by reversing mono-ubiquitylation and thus regulates several crucial cancer-related signaling pathways [20]. Ubiquitin-Specific Protease 13 (USP13), another member of deubiquitinating enzymes (DUBs) superfamily, has been reported to deubiquitinate and stabilize PTEN and thus suppresses tumorigenesis and glycolysis in breast cancer [22]. Although mounting studies suggest the oncogenic or tumor suppressor role of USP13 is context-dependent, however, its biological function in bladder cancer has not been reported yet.

Nuclear factor-kappa B (NF-kB) family has been well known as a hallmark in inflammation, immunity, cell survival and oncogenesis [23]. In our earlier studies, we have reported the functional role of miR-130b in human bladder cancer. It is not only directly upregulated by NF-kB but also capable of sustaining the constitutive activation of NF-kB by inducing the deubiquitylation of NF-kB subunits p50 and p65 [24]. MiR-130b belongs to the miR-130 family, which is composed of four members: miR-130a, miR-301a and miR-130b/301b cluster. The miR-130 family has been identified as a pan-cancer onco-miR family by targeting a set of tumor suppressor genes [25]. In the present study, we reported a novel biological function of miR-130b/301b cluster in facilitating bladder cancer progression. Our data revealed that knockdown of USP13 by short hairpin RNA (shRNA) significantly decreased PTEN protein expression in BC cells. Meanwhile, silencing of USP13 greatly enhanced proliferation, invasion and migration of the tested cells. USP13 was predicted and verified to be a common target of both miR-130b-3p and miR-301b-3p, and its expression could be reduced by NF-kB-enhanced transcription of miR-130b~301b cluster. Furthermore, restoration of USP13 rescued PTEN expression, cell phenotypes and xenograft tumor growth which is altered by NF-kB activation. Here, we reported that NF-kB/miR-130b/301b cluster axis promoted loss of PTEN expression by decreasing USP13 expression and enhanced progression of bladder cancer. Reintroduction of USP13 could block the pathway and rescue PTEN protein expression, and thereby inhibited the carcinogenesis of bladder cells.

## Methods

### Tissues

For the use of clinical materials for research purposes, prior patients’ written consent and approval were obtained from the China Medical University and The First Affiliated Hospital of China Medical University. A total of 50 patients with bladder urothelial cell carcinoma underwent partial cystectomies or radical cystectomies from 2014 to 2016 at the Department of Urology of the First Affiliated Hospital of China Medical University in China (Table 1). The tissue specimens were harvested and then immediately frozen in liquid nitrogen and stored at − 80 °C. Histologically, the tumors were classified according to the 2004 World Health Organization histologic classification of urinary tract tumors, and were staged using the 2002 American Joint Committee on Cancer system.

**Table 1:** The clinicopathological characteristics in 50 patients with bladder cancer

Parameters	Number of cases
Age	
Age ≥ 65, N(%)	36(72)
Gender	
Male sex, N(%)	35(70)
Histological grade	
High grade, N(%)	34(68)
Muscle invasion	
Positive, N(%)	38(76)
Distant metastasis	
Positive, N(%)	5(10)
Lymphatic invasion	
Positive, N(%)	8(16)

### Cell culture

The human bladder carcinoma cell lines, 5637 and UM-UC-3 cells were purchased from the cell bank of Chinese Academy of Sciences (Shanghai, China). These cells are maintained in RPMI 1640 (HyClone, Logan, UT, USA) supplemented with 10% FBS (HyClone) and 1% penicillin-streptomycin (HyClone) at 37 °C, and regularly tested to ensure that they are mycoplasma-free.

### RNA isolation and real-time quantitative PCR

Total RNA, including micro-RNA from cultured cells and fresh surgical bladder tissues, was extracted using a miRNeasy™ Mini Kit (Qiagen), according to the manufacturer’s instructions. cDNA synthesis and quantitative real-time PCR were performed using a mercury LNA™ Universal RT microRNA PCR kit (Exiqon, Skelstedet, Vedbaek, Denmark). The hsa-miR-130b-3p, has-miR-301b-3p and U6 LNA™ PCR primer sets were purchased from Exiqon. qRT-PCR was performed using SYBR® Premix Ex Taq™ (Tli RNaseH Plus; Takara Biotechnology CO. LTD., Dalian, China) and LightCyclerTM 480 II system (Roche, Basel, Switzerland). β-actin and U6 snRNA were used as endogenous controls for mRNA and miRNA, respectively. The primers used to amplify the target genes are listed in Additional file 5: Table S1. The relative levels of expression were quantified and analyzed using LightCyclerTM 480 software 1.5.1.6.2 (Roche, Basel, Switzerland). The 2-ΔΔCT method was performed to calculate the relative expression, and expression levels of negative controls were used for calibration. Three independent experiments were performed to analyze the relative gene expression.

### Luciferase reporter assay

Luciferase reporter assay was performed using a Dual Luciferase Reporter Assay Kit (Promega) according to the manufacturer’s protocol. The luciferase reporters, psiCHECK2-USP13 wild type and psiCHECK2-USP13 mutant type were synergized by purchased from GenePharma (Shanghai, China). The luciferase reporter construct was co-transfected with agomir of miR-130b-3p and miR-301b-3p into the 5637 cells by Lipofectamine 3000 (Invitrogen) according to the manufacturer’s guidelines. The relative luciferase activity was measured by Synergy HTX multi-mode microplate reader (BioTek).

### Western blotting analysis

Antibodies to USP13 (ab99421), NF-kB p65 (ab16502), NF-kB phosph-p65 (ab86299), pan-AKT (ab8805), phosph-AKT (Ser473) (ab8932), phosph-AKT (Thr308) (ab8933), PTEN (ab170941) and GAPDH (ab181602) (all from abcam) were used according to the manufacturers’ protocols. The western blotting analysis was performed as described before. Briefly, equal amounts of protein extracts were separated by 10% SDS-polyacrylamide gel electrophoresis (SDS-PAGE) and transferred to polyvinylidene fluoride (PVDF) membranes (Millipore, Billerica, MA, USA). The membranes were blocked with Tris-buffered saline plus Tween-20 (TBS-T; 0.1% Tween-20) with 5% (w/v) non-fat dry milk and were then incubated with primary antibodies in TBS-T at 4 °C overnight. After three washes with TBS-T for 15 min each, the membranes were incubated with the appropriate HRP-labeled secondary antibodies for 1 h at 37 °C. The immunobands were visualized using the ECL reagents (Transgen Biotechnology, Beijing, China) on a MicroChemi Chemiluminescent Imaging System (DNR Bio-Imaging Systems, Mahale HaHamisha, Jerusalem, Israel). The densitometric values for each band were calculated by Image J 1.46r software (Wayne Rasband, National institutes of Health, Bethesda, MA, USA), and the statistical analysis were conducted based on the ratios of target protein/GAPDH.

### Plasmids and transfection

The Flag-vector, Flag-USP13, pLvx-USP13, pLvx-p65, pLvx-PTEN and relative pLvx-vector were constructed by GenePharma (shanghai, China). shRNAs were constructed using pLKO.1 vector according to Addgene TRC Cloning Protocol. The micro RNA agomir and antagomir were synergized by and purchased from GenePharma (Shanghai, China). Transfections were performed using the Lipofectamine 3000 Reagent (Invitrogen) following the manufacturer’s protocol. Final concentrations for miRNA agomir or plasmids were 50 nM and 0.75 μg/ml. Cells were cultured in a six-well plate with 2 ml culture medium. The concentration for lentivirus transduction was 5 × 10^6^ transducing units of lentivirus. The stable cell lines were constructed using puromycin (200 μgml− 1).

### Proliferation assay

The cell proliferation capacity was evaluated by a Cell Counting Kit-8 (CCK-8) (Dojindo, Tokyo, Japan) and a cell colony formation assay, according to the manufacturer’s protocol. The absorbance value was measured at 450 nm to determine cell viability using a 96-well plate reader (model 680, Bio-Rad, Hertfordshire, UK). For cell colony formation assay, the cells were plated in 6-well plates (300 cells per well), and after 24 h, cells were subject to indicated treatments, and followed by 10- days incubation in complete medium. Colonies were fixed with 10% formaldehyde for 10 min and stained with 1.0% crystal violet for 5 min, and images were captured. The number of colonies, defined as > 50 cells/colony, was counted.

### Transwell assay

The transwell assay was performed to evaluated cell invasive and migrative capacities using the transwell (Corning) with or without matrigel (BD Biosciences), respectively, according to the manufacturer’s instructions. Cells were re-suspended in RPMI 1640 containing 1% FBS, and 0.2 ml cell suspension (1 × 10^4^/ml) was seeded into the top chamber, whereas 0.6 ml of RPMI 1640 containing 10% FBS was filled in the lower chamber as the chemoattractant. After 24 h of incubation at 37 °C with 5% CO2, the number of cells invading or migrating to the lower chamber was counted in 10 randomly selected visual fields, and the images were captured by a Leica DM3000 microscope (Leica).

### Cell apoptosis by flow cytometry

miR-130b/301b overexpressing or USP13 knockdown 5637 cells (3 × 104 per well) were seeded into 24-well culture plates and cultured for 24 h. Then, the cells were harvested, washed three times in phosphate buffered saline (PBS), and resuspended in 0.4 ml of ice-cold PBS. The resuspended cells were incubated with propidium iodide (PI) and a fluorescein isothiocyanate (FITC)-conjugated monoclonal antibody specific for Annexin V (BD, San Diego, CA, USA). The results were measured by flow cytometry (Becton Dickinson Biosciences, San Jose, CA), and the data was analyzed by the ModFit LT software package. The experiments were performed independently in triplicate.

### Immunohistochemistry

Clinical pathological sections of bladder cancer tissue specimens from thirty bladder cancer patients were provided by Department of Pathology at the First hospital of China Medical University. The expression of USP13 and PTEN was detected using an UltraSensitive™ SP (Mouse/Rabbit) IHC kit (Maxin-Bio, Fuzhou, Fujian, China) according to the manufacturer’s instructions. Briefly, sections were firstly dewaxed in xylene and ethanol, and antigen retrieval was performed using a microwave for 10 min at 100 °C. The sections were then incubated with antibodies for 1 h, followed by biotinylated anti-IgG antibody and streptavidin-biotinylated-complex horseradish peroxidase. DAB and hematoxylin were used for nuclear staining. The images were then captured by upright metallurgical microscope (Olympus, Tokyo, Japan) under an original magnification of 200×. Scoring was done by two pathologists who counted the intensity of positive cells in a defined area. To quantify NF-kB p65, USP13 or PTEN staining, staining intensity was classified as: 0 (no staining), 1 (low staining), 2 (medium staining) and 3 (high staining). 0 and 1 were defined as low expression, and 2 and 3 were defined as high expression. USP13 and PTEN expression was classified as USP13-low or USP13-high and PTEN-low or PTEN-high, respectively. Fisher’s exact test was used to correlateUSP13 and PTEN staining.

### Immunoprecipitation assay

For immunoprecipitation, cells were harvested in lysis buffer containing 50 mM Tris-HCl pH 7.5, 150 mM NaCl, 0.5% NP40, 1 mM EDTA, 1 mM sodium orthovanadate, and a 1× protease inhibitor cocktail. To immunoprecipitate Flag-tagged proteins, lysates were firstly incubated with anti-Flag M2 beads (Sigma) overnight, beads were then washed three times, and precipitated proteins were eluted with SDS loading buffer.

### Preparation of UM-UC-3 cells for in vivo experiment

UM-UC-3 cells were transduced with a lentiviral control vector or a construct harboring human NF-kB p65. Simultaneously, NF-kB p65-expressing cells were transduced with either a lentiviral vector encoding USP13 (NF-kB p65 + USP13) or PTEN (NF-kB p65 + PTEN). Four types of UM-UC-3 cells (vector, NF-kB p65, NF-kB p65 + USP13, NF-kB p65 + PTEN) were used for the orthotopic tumor model and the metastasis model.

### Animal experiments

BALB/c nude mice (4–6 weeks old, 14–16 g) were purchased from Beijing Vital River Experimental Animal Technology Co. Ltd. and housed in department of laboratory animal science of China Medical University. The study was approved by Medical Laboratory Animal Welfare and Ethics Committee of China Medical University and the methods were carried out in accordance with the approved guidelines. The mice were randomly divided to groups (*n* = 5 for each group). The cells were separately injected into the flanks of athymic nude mice to establish a xenograft tumor. Tumors were examined twice weekly; the length, width, and thickness were measured with calipers, and tumor volumes were calculated using the equation (Length × Width^2^)/2. 50 days after injection, the animals were euthanized, and the tumors were excised, weighed, paraffin-embedded and subjected to staining assays.

For metastasis experiments, prepared 1 × 106 UM-UC-3 cells in 0.1 ml PBS were injected into the tail vein of 20 five-week-old female BALB/c nude mice which were randomly divided into four groups (*n* = 5 for each group). After 50 days of injection, the animals were euthanized, and the intact lung tissues were isolated from the mice. The tissue sections were stained with hematoxylin and eosin. The numbers of metastatic cancer nests were counted at 10 × 10 magnifications using an inverted microscope (Leica DM3000).

### Statistical analysis

Data are shown as mean ± SEM from at least three independent experiments. Statistical analyses involved Student’s t-test, One-way ANOVA, Fisher’s exact test and Kaplan-Meier analysis with SPSS 22 (IBM Corp., Armonk, NY) or GraphPad Prism 6 (GraphPad Software, Inc., La Jolla, CA). *P* < 0.05 was considered statistically significant.

## Results

### USP13 is a common target of mR-130b-3p and miR-301b-3p, and is down-regulated in human bladder cancer tumors

We previously reported that miR-130b acts as an oncogene in BC, and is directly regulated by NF-kB via binding with the gene promoter. And miR-130b is capable of maintaining activation of NF-kB by decreasing the expression of CYLD, which is a deubiquitylation enzyme and functions as a NF-kB inhibitor [24]. MiR-130b belongs to the miR-130 family, which is composed of four miRNA precursor genes: miR-130a (at chr11), miR-301a (at chr17), miR-130b and miR-301b (at chr22). The stem-loop precursors of miR-130b and miR-301b are coded 327 bp apart at chr22 (Additional file 1: Figure S1a), and they constitute as clustered miRNAs. The mature RNAs generated from miR-130b~301b cluster are hsa-miR-130b-3p and hsa-miR-301b-3p (hereafter referred as miR-130b and miR-301b, respectively), which share a common seed sequence. The miR-130b/301b cluster belongs to a pan-cancer oncogenic miRNA superfamily, and has been identified to target critical tumor suppressor genes [25]. Since we have discovered the tumorigenic role of miR-130b in BC, we further sought to explore the regulatory functions of miR-130b/301b cluster.

**Fig. 1:**
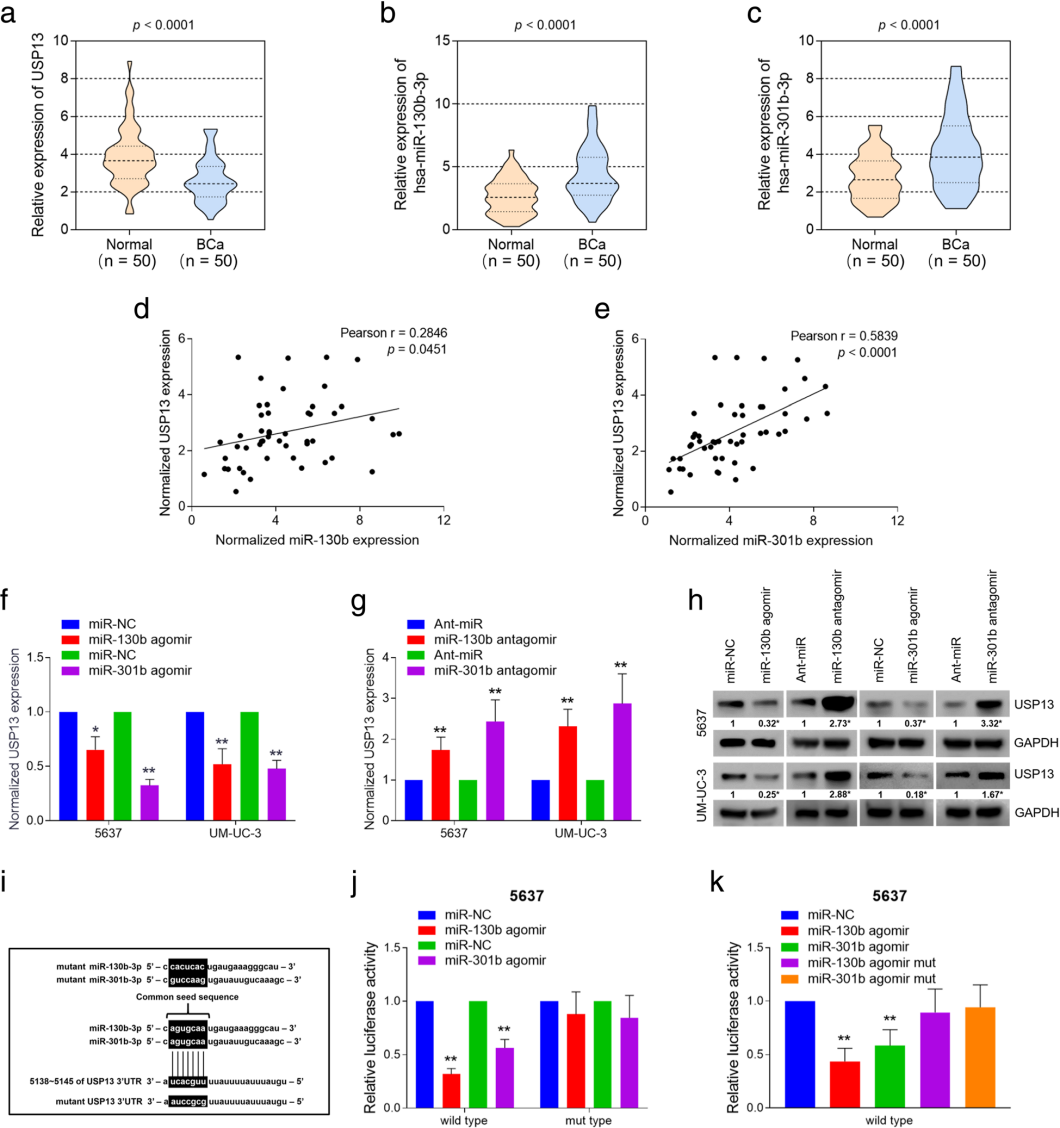
USP13 is a common target of miR-130b/301b, and expression of USP13 is significantly elevated in BC tissues. Expression of USP13 (**a**), miR-130b-3p (**b**) and miR-301b-3p (**c**) was detected in 50 pairs of normal bladder mucosa tissues and bladder cancer tissues by real-time PCR analysis. **d** and **e**. Pearson’s correlation coefficient test was performed to evaluate the correlation between expression of USP13 and miR-130b-3p/miR-301b-3p in 50 bladder cancer tissue specimens. The Transcriptional (**f** and **g**) and translational (**h**) levels of USP13 were measured in miR-130b/301b overexpressed or knocked down BC cells by real-time PCR analysis and western blotting analysis, respectively. **i.** The common seed sequence of miR-130b-3p and miR-301b-3p, and the miRNA response element on the 3’UTR region of USP13 gene. The mutations for USP13 3’UTR and agomir of miR-130b/301b were listed. **j.** The luciferase activity was measured in miR-130b/301b overexpressed 5637 cells co-transfected with luciferase construct harboring wild type 3’UTR or mutant type 3’UTR of USP13 gene. **k.** Luciferase activity was measured in 5637 cells co-transfected with luciferase construct harboring wild type 3’UTR of USP13 and miR-130b/301b agomir or mutant miR-130b/301b agomir. For real-time PCR, β-actin and U6 snRNA were used as the internal control for mRNA and miRNA, the Ct values for each group were compared using the the 2-ΔΔCt method. *P < 0.05 and **P < 0.01, as determined by Student’s T-test

Using bioinformatic analysis software (Targetscan [26] and miRDB [27]), we predicted several potential target genes of miR-130 family which display as tumor suppressor genes or crucial mediators in cancer-related pathways. Among them, we focused our attention on USP13 because of its potential role as a tumor suppressor by sustaining PTEN protein stability. Moreover, PTEN loss has been reported in human cancers previously and its loss is associated with constitutive NF-kB activation [28]. Thus, we sought to find if USP13 could be targeted by NF-kB/miR-130b~301b signaling and further facilitate the loss of PTEN. To investigate the regulatory role of USP13 in BC, we detected mRNA expression of USP13 in a cohort of 50 pairs of bladder cancer tissues and matched normal bladder mucosa tissues. The expression of miR-130b/301b was detected respectively as well. Results showed that expression of USP13 was significantly decreased in BC tissues compared with matched non-tumorous tissues (Fig. 1a). In contrast, the presence of miR-130b/301b was notably elevated in BC tissues (Fig. 1b and c). We next analyzed expression of USP13 gene and miR-130b/301b based on the data from The Cancer Genome Atlas (TCGA) database by starBase [29] and UALCAN [30] software. The evidence collected from TCGA-BLCA further supported our findings (Additional file 1: Figure S1b~S1e). In attempt to analyze the correlation between the expression of USP13 and miR-130b/301b in BC tissues, we performed Spearman’s correlation coefficient analysis based on the experimental results from our clinical cohort. Interestingly, we found USP13 expression was inversely correlated with the expression of miR-130b and miR-301b, respectively (Fig. 1d and e).

Next, we respectively overexpressed or knocked down miR-130b/301b in two BC cell lines, 5637 and UM-UC-3. 5637 (Grade I) and UM-UC-3 (Grade III) are two cell lines from primary urinary bladder cancer, and are commonly used as non-muscle invasive or muscle invasive bladder cancer models, respectively [31]. qRT-PCR (Fig. 1f and g) and western blot (Fig. 1h) analysis were performed to measure the alteration of USP13 expression. Results showed that overexpression of miR-130b/301b significantly decreased USP13 expression in transcription and translational levels, and knockdown of miR-130b/301b revealed an opposite effect on USP13 expression. The dual-luciferase reporter assay was then performed to assess the binding of miR-130b/301b with the 3’UTR region of USP13 mRNA. The results showed overexpression of miR-130b/301b notably inhibited the transcription of reporter construct containing wild type 3’UTR sequence of USP13 gene, whereas did not significantly affect the transcription of the control construct bearing the mutant 3’UTR of USP13 gene (Fig. 1i and j). We then employed the miR-130b/301b “mutant” agomir with the mutant seed sequence as a negative control, and respectively co-transfected the test cells with reporter plasmid bearing wild type USP13 3’UTR containing miR-130b/301b agomir or miR-130b/301b mutant agomir (Fig. 1i). Results revealed that the mutant of miR-130b/301b seed sequence blocked the binding of miR-130b/301b with the 3’UTR of USP13 gene, therefore abolished the inhibition of miR-130b/301b for the transcription of reporter gene (Fig. 1k). Taken together, USP13 is a target of miR-130b/301b and its expression decreased in BC tissues. Furthermore, we found decreased expression level of USP13 is associated with an increased risk of progression to BC, suggesting a tumor suppressor role of USP13 in bladder cancer.

### The biological functions of USP13 and miR-130b/301b in vitro

**Fig. 2:**
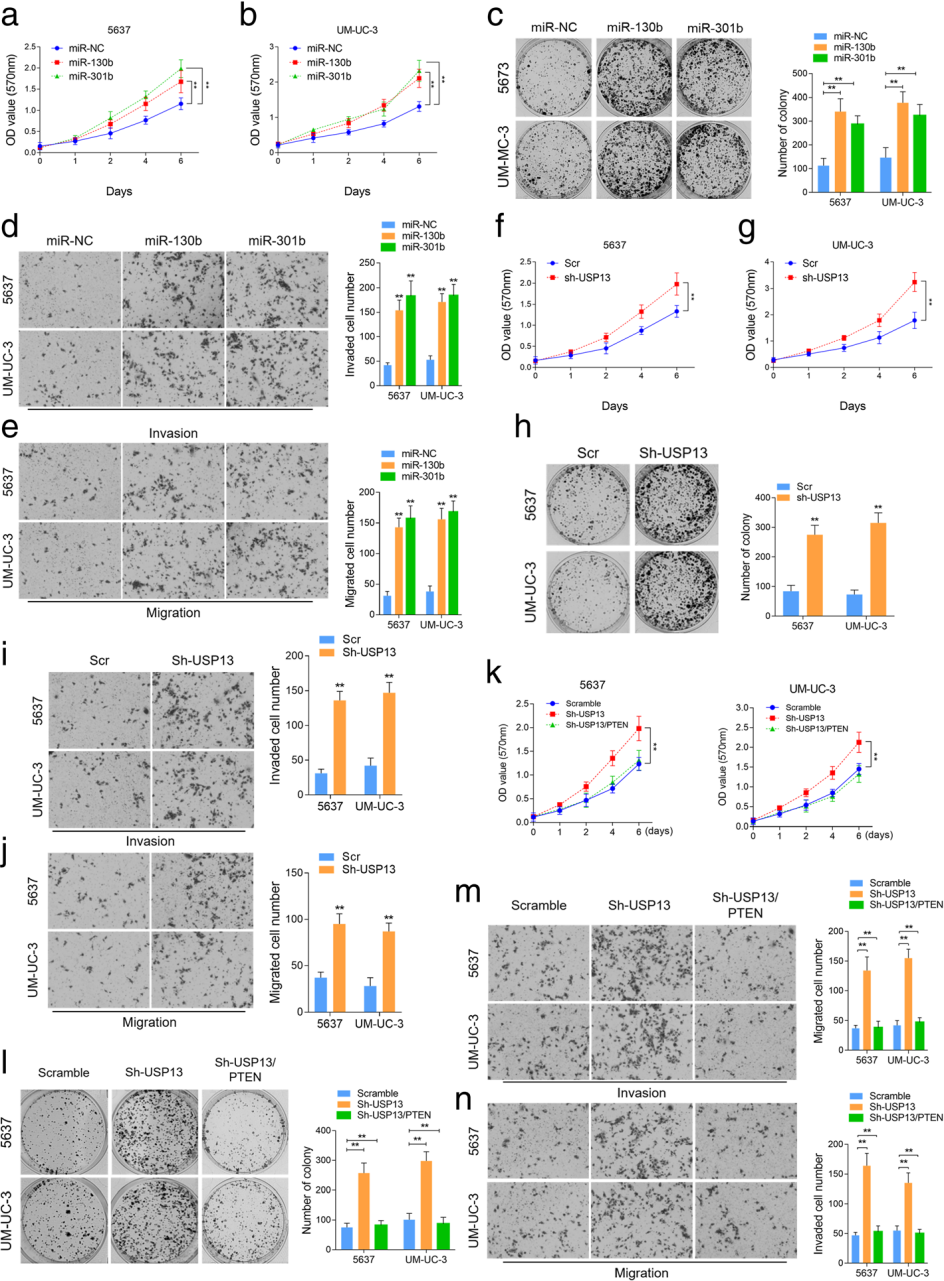
The biological function of miR-130b/301b and USP13 in vitro. Cell proliferative capacity was measured by Cell Counting-Kit 8 (CCK-8) (**a** and **b**) assay and colony formation assay (**e**) in miR-130b/301b overexpressed 5637 and UM-UC-3 cells. CCK-8 (**c** and **d**) and colony formation assay (**f**) were also performed to evaluate the cellular proliferation in USP13 knocked down 5637 and UM-UC-3 cells. Cell invasive and migrative capacities were measured by transwell assay in miR-130b/301b overexpressed (**g** and **h**) or USP13 knocked down (**i** and **j**) 5637 and UM-UC-3 cells. Cell proliferation was detected by CCK-8 (**k**) and colony formation assay (**l**) in USP13 knocked down alone or USP13 knocked down as well as PTEN expression restored 5637 and UM-UC-3 cells. Cell invasive (**m**) and migrative (**n**) capacities were measured by Transwell assay in USP13 knocked down alone or USP13 knocked down as well as PTEN expression restored 5637 and UM-UC-3 cells. Original magnification: 400×. *P < 0.05 and **P < 0.01, as determined by Student’s T-test

The in vitro phenotypic impact of miR-130b or miR-301b was assessed in BC cell lines, 5637 and UM-UC-3. A remarkable increase in cell proliferation was observed by cell counting kit-8 (CCK-8) (Fig. 2a and b) and colony formation assays (Fig. 2c) in miR-130b/301b overexpressed BC cells, whereas knockdown of miR-130b/301b significantly suppressed cell proliferation (Additional file 2: Figure S2a, S2b, S2c and S2d). Moreover, results of transwell assay displayed a significant enhancement in cell invasive and migrative capacities in response to miR-130b/301b overexpression (Fig. 2d and e), in contrast, miR-130b/301b knockdown remarkably reduced the number of invaded and migrated cells (Additional file 2: Figure S2e and S2f). To study the biological function of USP13 in bladder cancer, we overexpressed USP13 by transducing the BC cells with lentivirus vector encoding USP13 (Additional file 2: Figure S2 g). However, colony formation assay and transwell assay revealed no significant changes in cell proliferation, invasion and migration (Additional file 2: Figure S2 h and S2i). In contrast, USP13 knockdown by shRNA dramatically improved proliferation along with invasion and migration of the BC cells (Fig. 2f, g, h, i and j). We also tested the impact of miR-130b/301b or USP13 on cell apoptosis, however no significant difference in cell apoptosis was observed in response to miR-130b/301b overexpression or USP13 knockdown (Additional file 2: Figure S2j and S2 k). These findings suggested that USP13 might exert its anti-tumor function by interacting with tumor suppressors and sustain its function. This is in consistent with previously reported findings that USP13 deubiquitinates and stabilizes PTEN protein thus suppresses tumorigenesis in human cancers. To test this hypothesis, we then re-overexpressed PTEN expression in USP13 knocked down BC cells (Additional file 2: Figure S2 l). Results suggested that cell proliferative, invasive and migrative capacities were rescued in response to PTEN re-introduction (Fig. 2k, l, m, n). This supported our idea that USP13 might play as a tumor suppressor by blocking PTEN downregulation.

### USP13 abolished miR-130b or miR-301b mediated PTEN loss by directly binding to PTEN protein

**Fig. 3:**
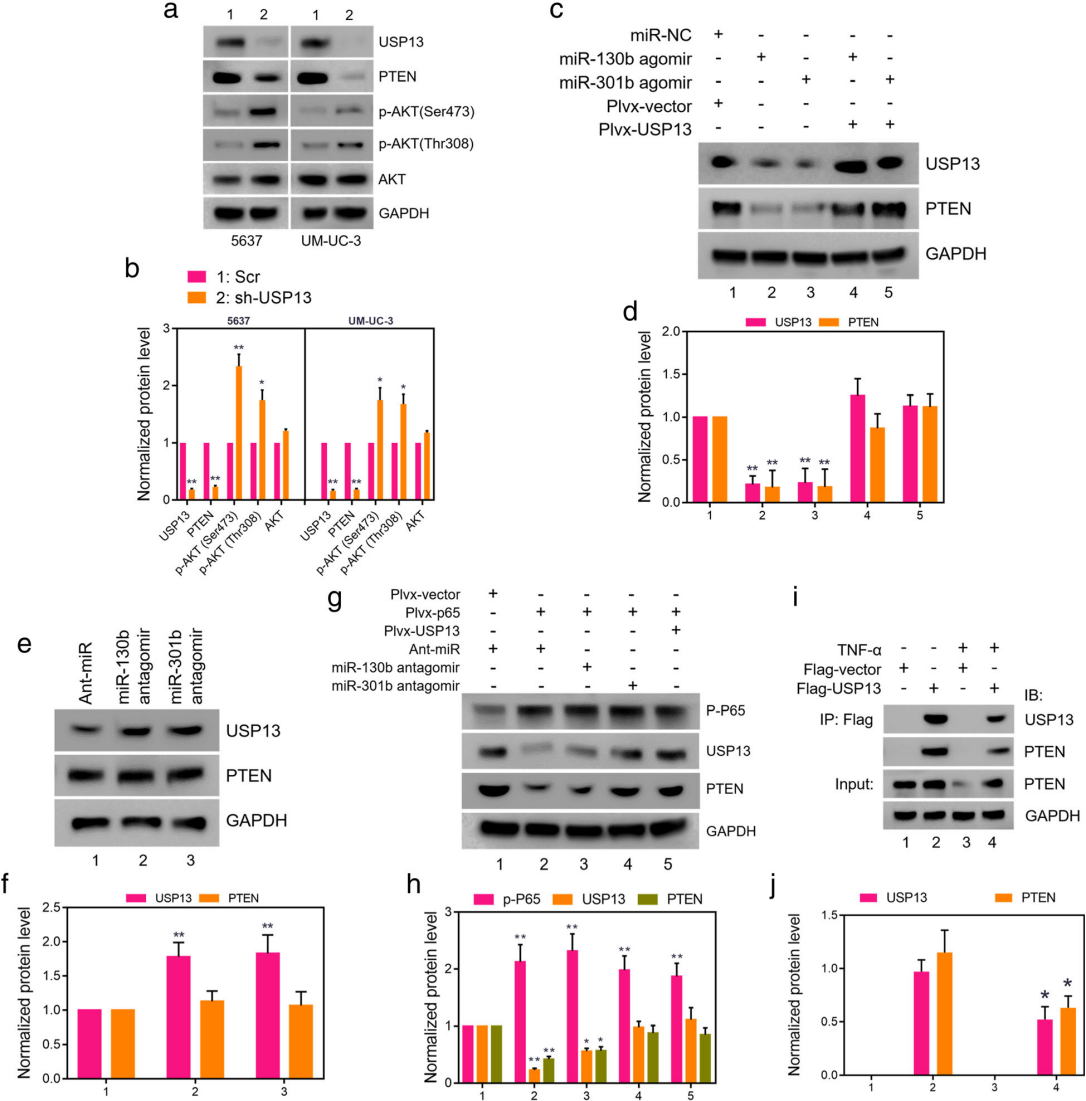
USP13 maintains PTEN expression through direct interaction. **a.** Expression of USP13, PTEN as well as phosphor-AKT and AKT were detected in USP13 knocked down 5637 and UM-UC-3 cells by western blot analysis, the band intensities were quantitated in **b**. **c.** agomir of miR-130b/301b was transfected into 5637 and UM-UC-3 cells, and USP13 expression was subsequently rescued. Western blotting analysis was performed to evaluate the expression of USP13 and PTEN, the band intensities were quantitated in **d**. **e.** miR-130b/301b was knocked down in 5637 and UM-UC-3 cells, and expression of USP13 and PTEN was detected by western blotting analysis, the band intensities were quantitated in **f**. **g.** NF-kB p65 was overexpressed in 5637 cells, and then agomir of miR-130b, miR-301b or pLvx-USP13 was transfected into the NF-kB p65 overexpressed cells, respectively. Expression of phosphor-p65, USP13 and PTEN was measured by western blotting analysis, the band intensities were quantitated in **h**. **i.** 293 T cells were transfected with Flag-tagged USP13 or empty vector for 24 h, then followed by TNF-a or relative vehicle treatment for 12 h. Lysates were subjected to immunoprecipitation with anti-Flag M2 beads. Bound proteins were analyzed by western blotting with USP13 or PTEN antibodies, the band intensities were quantitated in **j**. The internal control genes were GAPDH for western blot analysis and β-actin for real-time PCR analysis. The gels were run under the same experimental conditions. The band intensities were calculated by Image J 1.46r software, and the ratio of target gene to GAPDH was used to conduct the statistical analysis. *P < 0.05 and **P < 0.01, as determined by Student’s T-test

To investigate the functional role of USP13 in BC, USP13 expression was knocked down by two independent USP13 shRNAs (Additional file 3: Figure S3a and S3b) in 5637 and UM-UC-3 cells. Next, we analyzed the expression of PTEN, AKT, along with phospho-AKT (Ser473) and phospho-AKT (Thr308) proteins by western blot analysis. Results indicated that USP13 knockdown notably decreased PTEN protein expression and increased phosphor-AKT levels in BC cells (Fig. 3a and b). We then sought to explore whether miR-130b/301b could reduce PTEN expression via targeting USP13. Loss-of-function analysis was performed in 5637 and UM-UC-3 cells stably expressing USP13 and PTEN protein. Results showed that overexpression of miR-130b/301b dramatically decreased protein expression of USP13 and PTEN, while restoration of USP13 completely reversed the effect (Fig. 3c and d). In addition, no alteration of PTEN expression was observed in USP13-overexpression cells (Fig. 3e and f). This helps explain our previous finding that USP13 overexpression exerts no significant influence on BC cells phenotype.

We have demonstrated in our last study that NF-kB enhances transcription of miR-130b by binding to the promoter, and we have identified the Kappa-B binding site within the promoter region of miR-130b precursor by chromatin-immunoprecipitation (Chip) assay (Additional file 3: Figure S3c). The genomic structure of miR-130b and miR-301b suggested that the cluster could be driven by the same upstream promoter. Hence, we predicted that NF-kB drove the transcription of miR-130b/301b cluster and enhanced the presence/expression of both miR-130b-3p and miR-301b-3p. To assess the possibility, we endogenously activated NF-kB signaling by stimulating the test cells with tumor necrosis factor (TNF)-alpha. Transcriptional levels of NF-kB downstream targets, namely, NFKB inhibitor alpha (NFKBIA), TNF alpha induced protein 3 (TNFALP3), X-linked inhibitor of apoptosis (XIAP), C-C motif chemokine ligand 5 (CCL5), C-X-C motif chemokine ligand 8 (CXCL8), were detected to confirm the activation of NF-kB signaling (Additional file 3: Figure S3d and S3e). In consistent with our hypothesis, expression of miR-130b/301b is significantly increased following TNF-alpha stimulation (Additional file 3: Figure S3f and S3 g). Furthermore, we overexpressed NF-kB by transducing the cells with lentivirus vector encoding NF-kB subunit p65 (pLVX-p65) (Additional file 3: Figure S3 h). As our prediction, miR-130b/301b was upregulated in response to p65 overexpression (Additional file 3: Figure S3i and S3j). Thus, miR-130b/301b cluster is a transcriptional target of NF-kB.

Given the function of miR-130b/301b-USP13 in regulating PTEN expression and the established role of miR-130b/301b as a downstream target of NF-kB signaling, we then asked whether NF-kB regulate PTEN protein expression through miR-130b/301b-USP13 axis. Loss-of-function analysis was performed to assess our hypothesis. We transfected the cells with NF-kB subunit p65 and measured NF-kB activation by detecting the nucleus p65 protein expression level. Results indicated that NF-kB p65 overexpression dramatically decreased both USP13 and PTEN expression, while depletion of miR-130b/301b partially rescued expression of USP13 and PTEN. In addition, restoration of USP13 completely reversed the effect of NF-kB p65 overexpression on reducing USP13 and PTEN expression (Fig. 3g and h). In addition, co-immunoprecipitation (IP) was then performed to verify the direct interaction between USP13 and PTEN proteins. We ectopically expressed flag-tagged USP13 in 5637 cells (Additional file 3: Figure S3 k) and detected PTEN protein in the anti-flag antibody pull-down protein products in western blot assay. Of note, TNF-alpha stimulation in flag-tagged USP13 expressed 5637 cells with reduced the PTEN protein level was detected in the pull-down proteins (Fig. 3i, j and Additional file 4: Figure S4). These results supported a role for USP13 in the NF-kB-driven PTEN downregulation and tumorigenesis of BC. More importantly, NF-kB induced PTEN loss is most likely to be controlled by USP13 protein to a large extent.

### USP13 is a downstream effector of NF-kB-miR-130b/301b induced tumorigenesis of BC

**Fig. 4:**
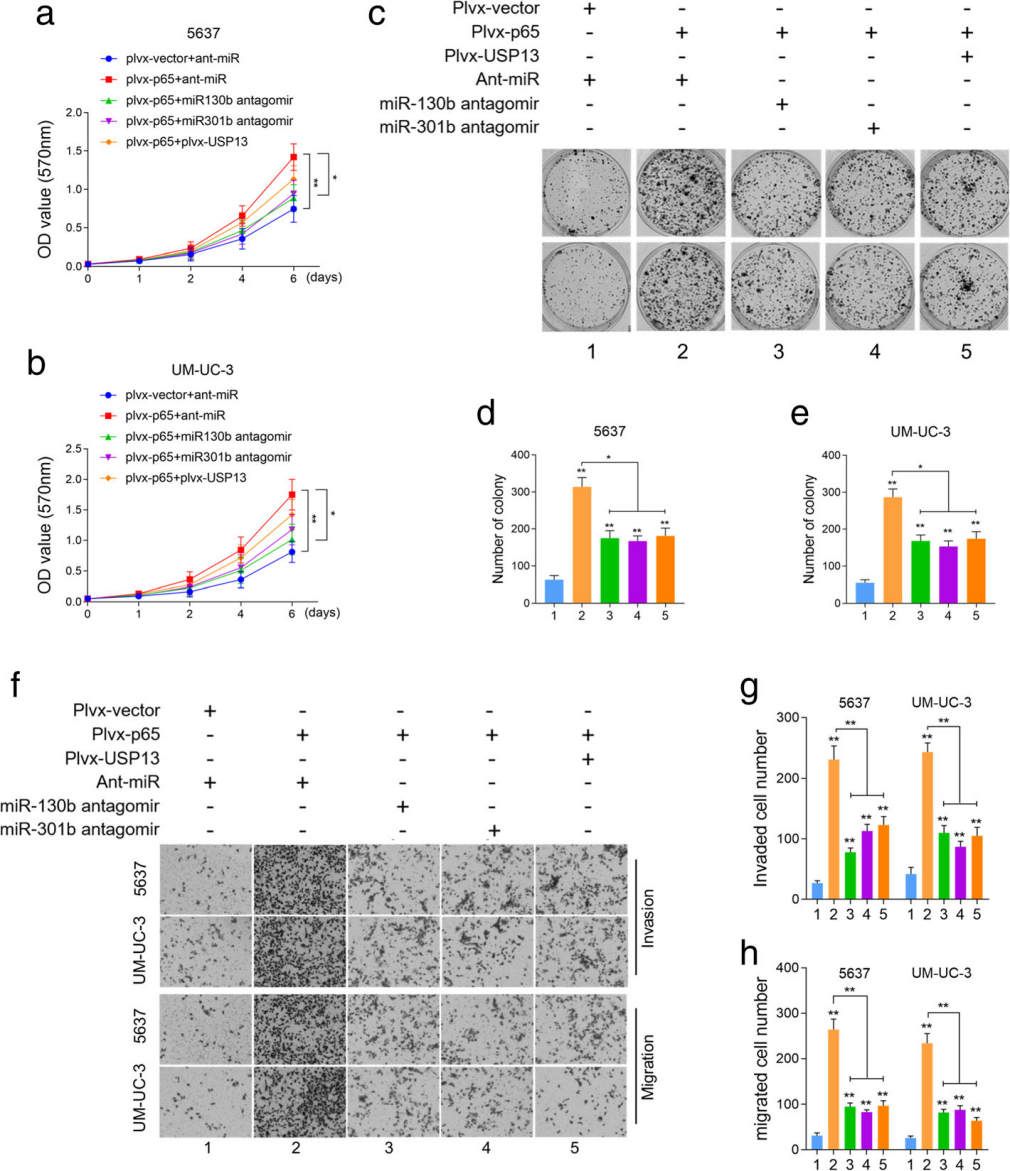
The biological function of NF-kB/miR-130b~301b/USP13 axis in vitro*.* **a-e.** NF-kB p65 was overexpressed in BC cells by transfecting with pLvx-NF-kB p65, then followed by transfection of antagomir of miR-130b/301b or USP13 overexpression. Cell proliferation index was determined by CCK-8 assay and cell colony formation assay. Each group was indicated as: 1 empty vector; 2 NF-kB p65 overexpression; 3 NF-kB p65 overexpression with miR-130b knockdown; 4 NF-kB p65 overexpression with miR-301b knockdown; 5 NF-kB p65 overexpression with restoration of USP13 expression. **f-h.** Cell invasive and migrative capacities were determined by Transwell assay. The cells were grouped as described in (**a**). Original magnification: 400×. *P < 0.05 and **P < 0.01, as determined by Student’s T-test

Having found that USP13 is capable of blocking NF-kB induced PTEN downregulation, we further attempted to verify whether USP13 functions as a tumor suppressor in NF-kB-driven tumorigenesis of BC. The in vitro phenotypic impact of NF-kB p65 overexpression was assessed in BC cell lines. CCK-8 (Fig. 4a and b) and colony formation assay (Fig. 4c, d and e) revealed the overexpression of NF-kB p65 dramatically increased cell proliferation and survival, while depletion of miR-130b/301b or restoration of USP13 partially rescued the enhancement of cell viability induced by NF-kB p65 overexpression. Transwell assay was then performed to assess cell invasive and migrative capacities. Results suggested that overexpression of NF-kB p65 greatly increased the number of migrated/invaded cells, however knockdown of miR-130b/301b or restoration of USP13 rescued the increased cell invasion and migration caused by NF-kB activation (Fig. 4f, g and h). These data supported our notion that USP13 suppresses tumorigenesis by blocking the NF-kB-driven PTEN downregulation in BC. Nonetheless, USP13 was not able to fully rescue the oncogenic function of NF-kB on BC cells, suggesting the possible involvement of other pathways in the process.

### Restoration of USP13 or PTEN partially rescued NF-kB promoted tumor formation and metastasis in vivo

**Fig. 5:**
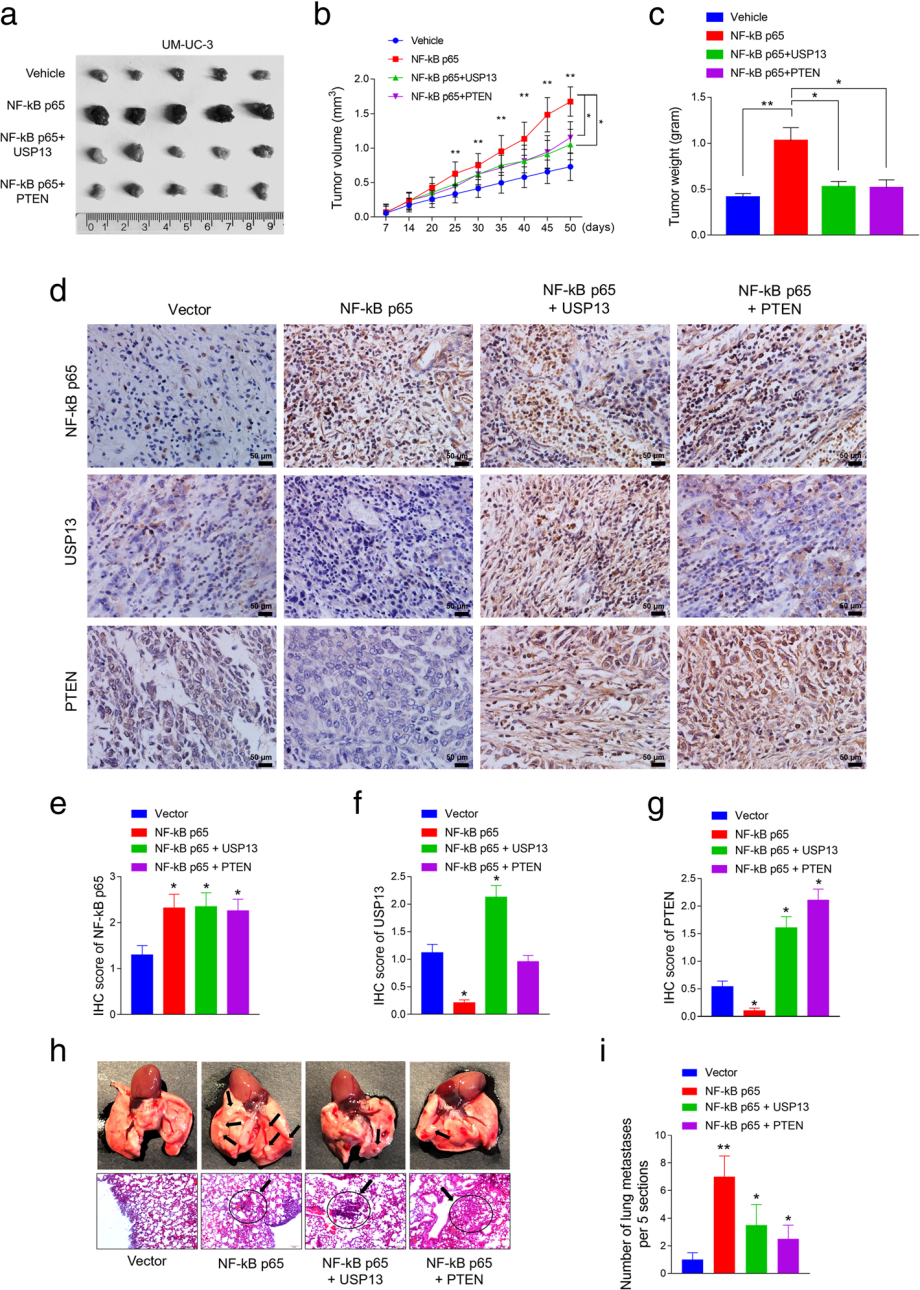
Re-expression of USP13 or PTEN partially rescued the tumor growth and metastasis in vivo. **a.** In vivo tumor lumps of xenograft mouse models composed of 4 types of UM-UC-3 cells: empty vector, NF-kB overexpression, NF-kB overexpression with USP13 re-expression, NF-kB overexpression with PTEN re-expression. Mice were sacrificed at the 50th day after injection and each tumor lump was removed from the body. **b.** The tumor growth curves of in vivo tumor volumes. Data are shown as mean ± s.d. of the tumor volumes, *n* = 5, *P < 0.05 and **P < 0.01 (ANOVA). **c.** The mean tumor weight of each group. Data are shown as mean ± s.d. of the tumor weights, *n* = 5. *P < 0.05, **P < 0.01 (ANOVA). **d.** IHC staining of NF-kB, USP13 and PTEN in xenograft tumors. The IHC scores were evaluated using a numerical score based on the number of positive cells in three different fields in each section, with a score of 0 indicating no positive cells; 1 indicating 10% positive cells; 2 indicating 10–50% positive cells; and 3 indicating 450% positive cells6. One section was evaluated for each sample, 5 samples/group. The IHC score for NF-kB p65, USP13 or PTEN was shown in **e, f** and **g**, respectively (*P < 0.05, Student’s t-test). **h.** Images of the lungs with metastatic nodules removed from the mice (**upper panel**). The lung metastasis nude mice model was conducted by injecting four types of cells (indicated in **a**) into the nude mice via the tail vein. Lungs were subjected to hematoxylin-eosin staining, and the numbers of metastatic nodules were captured and counted under the microscope (**h**: lower panel, **i**). The number of lung metastases per 5 sections for each group was conducted for statistical analysis (*P < 0.05 and **P < 0.01, ANOVA)

To evaluate the biological function of USP13 in vivo, UM-UC-3 cells were subcutaneously injected into nude mice. There were four groups based on the pretreatment of the cells: group 1 (vector) was injected with UM-UC-3 cells transduced with control lentivirus vector; group 2 (NF-kB p65) was injected with UM-UC-3 cells transduced with lentivirus construct harboring NF-kB p65; group 3 (NF-kB p65 + USP13) and group 4 (NF-kB p65 + PTEN) were respectively injected with UM-UC-3 cells transduced with lentivirus vector containing NF-kB p65 and either USP13 or PTEN. We found that tumor lumps in the NF-kB p65 overexpressing group were significantly larger and heavier than that in the control group. Furthermore, restoration of either USP13 or PTEN could partially reduce the tumor growth induced by NF-kB. On the 50th day, the mice were sacrificed and tumor lumps size and weight in each group were measured (Fig. 5a, b and c). The tumors were then subjected to staining assay (Fig. 5d). Expression of NF-kB p65, USP13 or PTEN proteins was measured by IHC staining assay and was quantified by classifying the staining intensity as 0 (no staining), 1 (low staining), 2 (medium staining) and 3 (high staining). Results suggested that the overexpression of NF-kB p65 significantly decreased the expression levels of USP13 and PTEN, and re-introduction of USP13 rescued PTEN expression (Fig. 5e, f and g).

To further examine the effect of USP13 on metastasis in vivo, the same four groups of engineered cells were injected into the tested mice via the tail vein. After 50 days of injection, the mice were sacrificed, and the lungs were removed and examined. We observed that the metastatic lesions at the surface of the lungs were more plentiful in the NF-kB overexpressing group than in the control group. Lung metastasis could also be observed in USP13 or PTEN restoration group, but the number was significantly decreased comparing with that of the NF-kB overexpressing group (Fig. 5h). The lungs were then subjected to hematoxylin-eosin staining, and the numbers of metastatic nodules were counted under the microscope. Consistent with the morphological characteristics, there were more metastatic lesions in the lungs of mice injected with NF-kB overexpression cells compared with the control group, and USP13 or PTEN re-introduction decreased the number of lung metastatic nodules comparing with that of the NF-kB overexpressing group (Fig. 5i).

### High PTEN level correlates with USP13 overexpression in human BC specimens

**Fig. 6:**
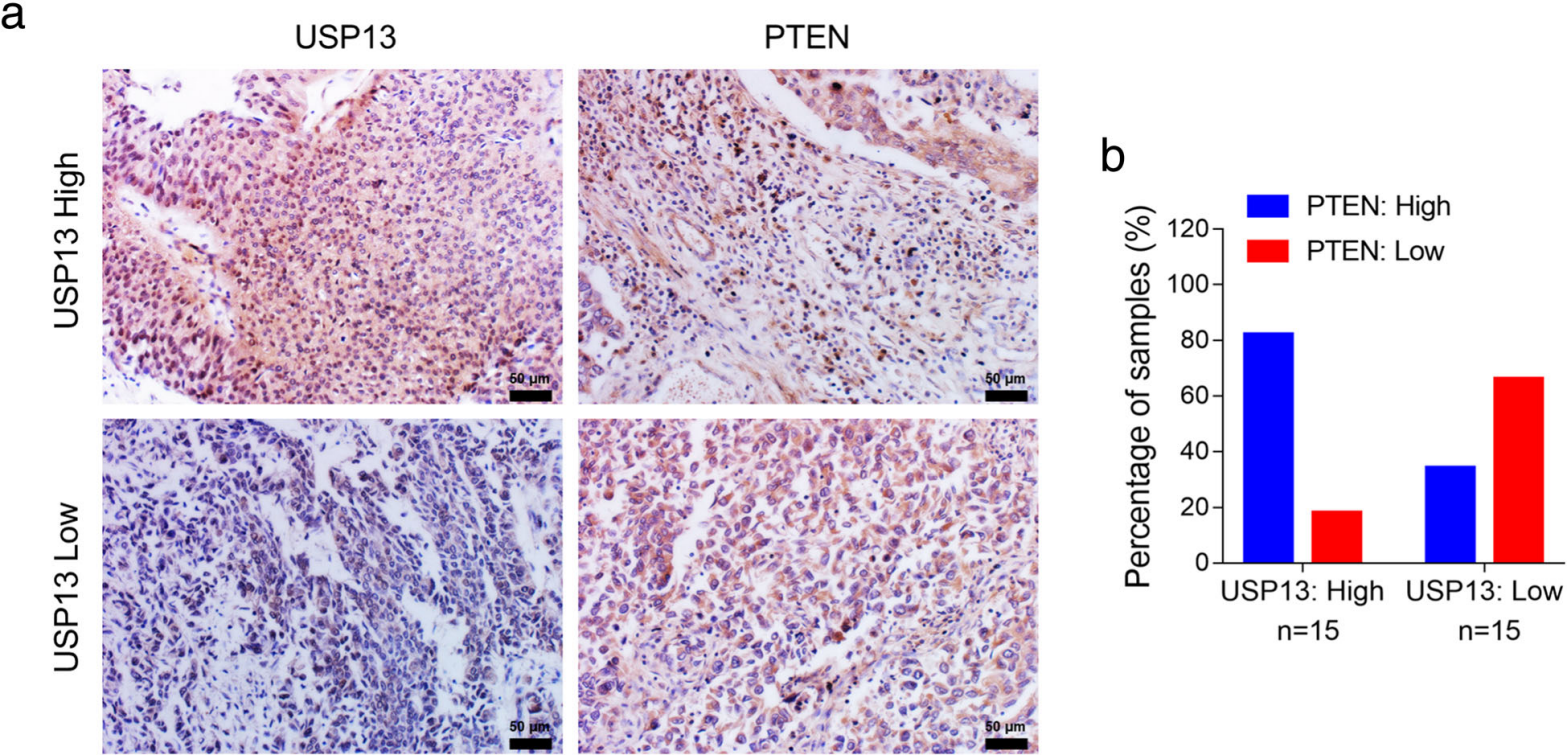
High PTEN levels correlate with USP13 overexpression in a subset of human bladder cancer tissue specimens. **a.** Representative images demonstrating PTEN and USP13 immunohistochemistry (IHC) staining of human bladder cancer tissue specimens from 30 bladder cancer patients. **b.** Quantification of PTEN or USP13 staining in BC tissue specimens. Staining intensity of PTEN or USP13 was scored as 0 to3 (0: no staining, 1: weak staining, 2: medium staining, and 3: strong staining. 0 and 1 were classified as low-expression, whereas 2 and 3 were defined as high-expression. High PTEN expression was correlated with high USP13 expression (*p* < 0.05, Fisher’s exact test)

To further demonstrate the correlation between expression of USP13 and PTEN, we performed IHC staining in 30 human BC specimens (Fig. 6a). Based on staining intensity, we defined USP13 samples as either USP13-low or -high. Likewise, PTEN staining was defined as either PTEN-low or -high. Analysis of USP13 and PTEN expression (Fig. 6b) suggested that USP13-high was positively correlated with PTEN-high (*p* < 0.05, Fisher’s exact test). The above data again supported the potential association of USP13 and PTEN in human bladder tumors.

## Discussion

Our present study revealed a critical role of USP13 in the tumorigenesis of BC through interacting with PTEN protein and blocking NF-kB-driven PTEN downregulation. USP13 knockdown in BC cells promoted their proliferation, invasion and migration. USP13 was predicted and verified to be a target of miR-130b/301b. Overexpression of miR-130b/301b exhibits similar malignant phenotypic alteration in BC cells as knockdown of USP13 does. Activation of NF-kB signaling increased the presence of both miR-130b and miR-301b, and reduced expression level of USP13 protein as well as PTEN. Moreover, reintroduction of USP13 repressed the signaling transduction of NF-kB and largely rescued PTEN expression in NF-kB activated or miR-130b/301b overexpressed BC cells. Additionally, it can partially rescue the NF-kB-enhanced proliferation, invasion and migration of cancer cells in vitro and in vivo. Hence, we concluded that USP13 acted as a tumor suppressor in BC, a downstream effector of NF-kB signaling and a crucial regulator of PTEN protein.

PTEN is one of the best well-characterized tumor suppressors, that it governs a variety of cellular processes including survival, proliferation and energy metabolism by suppressing the PI3K/mTOR pathway through its lipid phosphatase activity [10, 11, 32]. Therefore, understanding/unraveling the mechanisms regulating PTEN expression and function, including transcriptional regulation, post-transcriptional regulation, post-translational modifications and protein–protein interactions, could have a significant impact on cancer initiation, progression and invasion. USP13 belongs to the deubiquitinating enzyme superfamily and has been indicated in tumorigenesis by deubiquitinating tumor suppressors p53 [33], PTEN [22] and MITF [34]. There have been several studies suggesting that the regulatory role of USP13 in human cancer could be controversial. Han el al reported that USP13 gene is amplified in serious ovarian cancers and its overexpression is correlated with poor clinical outcome, indicating a potential therapeutic role of USP13 in ovarian cancer [35]. Li et al. demonstrated that USP13 plays as regulator for DNA repair and overexpression of USP13 enhances ovarian cancer cells resistance to chemotherapy [36]. Study by Zhao et al. identified that USP13 stabilizes and overexpresses microphthalmia-associated transcription factor (MITF) and is essential for proliferation of melanoma cells [34]. Nonetheless, USP13 is reported to deubiquitinate and stabilize PTEN protein, and functions as a tumor suppressor in breast cancer [22]. Despite the context-dependent role of USP13, no evidence has yet been provided to clarify its function in bladder urothelial cancer. Our data for the first time revealed that USP13 plays a critical role in blocking NF-kB-mediated PTEN downregulation. Knockdown of USP13 by USP13 shRNA, miR-130b/301b overexpression or NF-kB activation in BC cells leads to the loss of PTEN expression, which was subsequently demonstrated to be reversed by reintroduction of USP13. Restoration of USP13 or PTEN significantly rescued the invasive cell phenotypes and xenograft tumor growth.

**Fig. 7:**
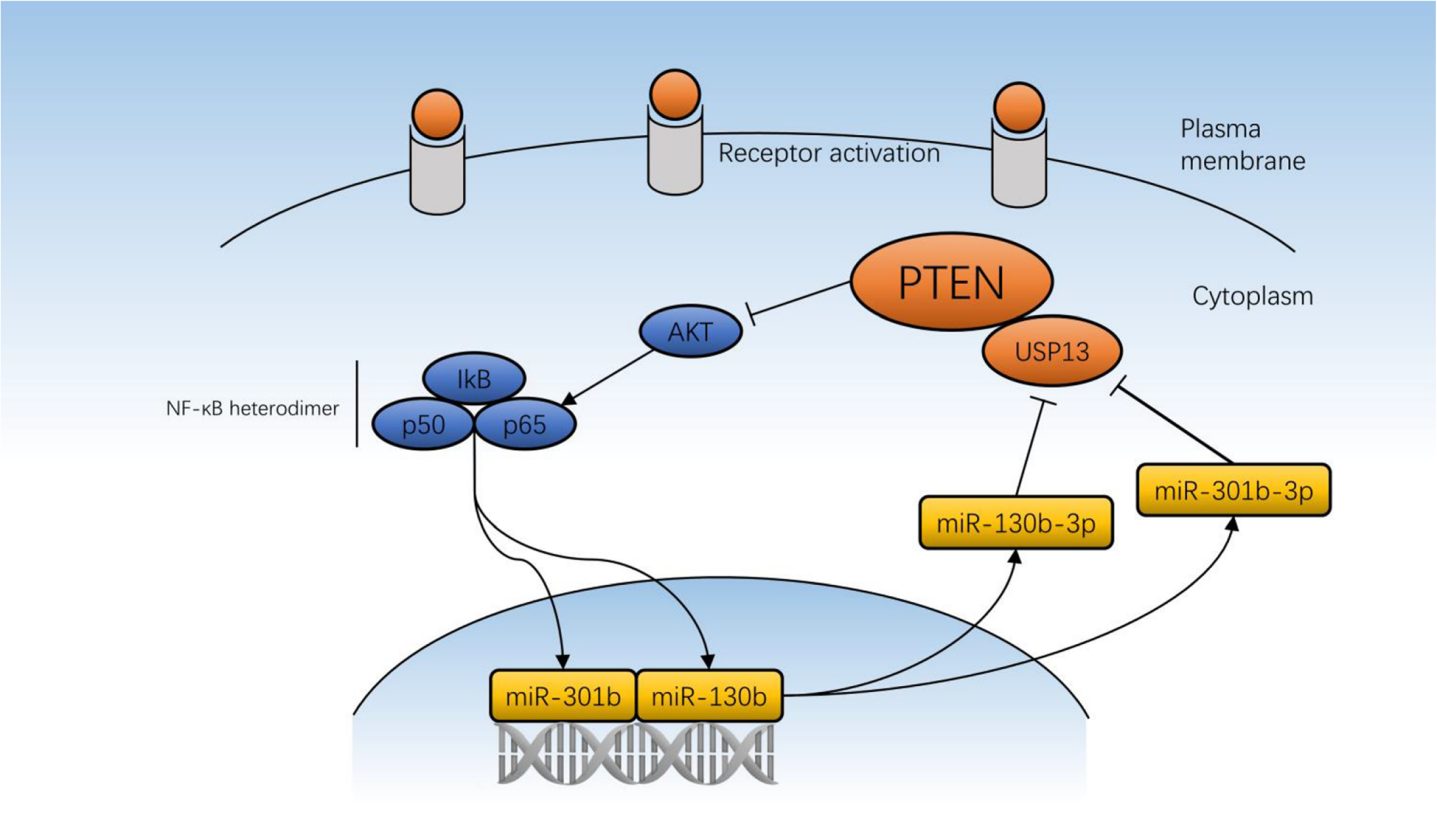
Mechanistic diagram illustrates the signaling transduction of NF-kB/miR-130b~301b cluster/USP13 axis. NF-kB decreases USP13 expression by inducing the transcription of miR-130b/301b, thereby suppresses expression of PTEN. On the other hand, PTEN governs the activity of NF-kB by inhibiting the PI3K/AKT signaling. The regulatory loop of NF-kB-induced loss of PTEN expression promotes constitutive activation of NF-kB and tumorgenesis of bladder cancer

NF-kB plays a critical role in preventing cell apoptosis, promoting tumor growth and activating of inflammatory response. PTEN functions as tumor suppressor by inhibiting PI3K/AKT/mTOR-mediated cell survival pathway. Notably, NF-kB activation has been identified to be necessary and sufficient for inhibition of PTEN expression [28, 37]. Mounting evidence has been demonstrated to clarify the mechanisms involving in the regulation of NF-kB for PTEN expression and function. NF-kB subunit p65 suppressed PTEN transcription by binding with its transcription co-activator CBP/p300 thus blocking the transcription activation of PTEN [37]. In Epstein-Barr virus infected nasopharyngeal carcinoma (EBV-NPC) cells, EBV latent membrane protein 1(LMP1) upregulates DNA methyltransferase 3b (DNMT3b) transcription in a NF-kB p65-dependent-manner, thereby resulting in relatively higher methylation intensity at PTEN CpG islands, and ultimately suppressing PTEN protein expression [38]. Interestingly, a recent study using the immortalized cell line of human urothelial cells (UROtsa), a well-established model to study carcinogenesis mechanisms of human bladder cancer, revealed that monomethylarsonous acid (MMAIII)-induced NF-kB activation modulates PTEN expression. Moreover, NF-kB subunit p50 binding to PTEN promoter is associated with decreased PTEN expression [39]. Our current work further enriches the regulatory network of NF-kB signaling for the post-transcriptional modulation of PTEN (Fig. 7).

The miR-130 family belongs to a pan-cancer oncogenic microRNA superfamily. It exercises its role as a tumor suppressor by negatively regulating the expression of target genes such as PTEN, TGFBR2, ZBTB4 and SMAD4 [25]. Here, we provided new evidence to demonstrate that miR-130 plays as a key regulator in sustaining constitutive and maximal activation of NF-kB through targeting DUBs that interrupt activation and signaling transduction of NF-kB. Our earlier study has revealed CYLD, a deubiquitinating enzyme as well as a strong NF-kB suppressor, was targeted by miR-130b and suppressed by NF-kB-mediated miR-130b overexpression. In this study, we showed that miR-130b/301b targeted USP13 and subsequently led to PTEN protein expression reduction. NF-kB requires PI3K- and Akt-dependent pathways to be fully activated by cytokines or growth factors, therefore down-regulation of PTEN is an essential step for maximal activation of NF-kB [40]. PTEN suppresses NF-kB activation by blocking PI3K/AKT pathway, thus inhibiting the nuclear translocation and DNA-binding potential of NF-kB subunits [41, 42]. A potential cross-regulatory loop could be established involving across NF-kB, miR-130b/301b cluster, USP13 and PTEN, which determines proliferation and survival of BC cells and tumorigenesis of BC (Fig. cartoon).

## Conclusions

In conclusion, the present study demonstrates the tumor suppressor role of USP13 in BC and the potential regulatory loop network of NF-kB/USP13/PTEN. USP13 has been identified to be a target of miR-130b/301b and a downstream effector of NF-kB. Overexpression of NF-kB subunit p65 reduces protein expression of USP13 and PTEN. Overexpression of miR-130b/301b or USP13 knockdown by shRNA delivery decreases USP13 expression, and further reduces PTEN protein expression and leads to enhancement of cell proliferation, invasion and migration. Overexpression of USP13 alone doesn’t affect cellular function and PTEN expression, however restoration of USP13 in NF-kB activated BC cells antagonizes the oncogenetic function of NF-kB, rescues PTEN expression and results in less aggressive cellular phenotypes. Moreover, NF-kB-mediated PTEN downregulation is very likely to be crucial for the full activation of NF-kB.

## Additional files

Additional file 1: **Figure S1**. a. The schematic diagram of mir-130b and mir-301b gene. b. Expression of USP13 in normal bladder tissues and bladder tumor tissues from TCGA dataset. c. Expression of USP13 in normal bladder tissue and bladder tumor tissues based on individual stages (TCGA). d and e. Expression of hsa-miR-130b-3p and hsa-miR-301b-3p in normal bladder tissue and bladder tumor tissue from TCGA dataset. (PDF 724 kb)

Additional file 2: **Figure S2**. Cell proliferation index was measured in BC cells after knockdown of miR-130b/301b by CCK-8 assay (a and b) and colony formation assay (c and d). Cell invasive and migrative capacities were measured in BC cells after knockdown of miR-130b/301b by transwell assay (e and f). Number of colonies and invaded/migrated cells was counted by Image J software. Original magnification: 400×. *P < 0.05 and **P < 0.01, as determined by Student’s T-test. g. USP13 was overexpressed by transducing the lentivirus vector encoded USP13 gene into the cells. h and i. USP13 overexpression alone doesn’t affect cell proliferation and invasion of bladder cancer cell line. j and *K. no* significant alterations were observed in cell apoptosis after miR-130b/301b overexpressing or USP13 knockdown in 5637 cells. i. 5637 and UM-UC-3 cells were transduced by Sh-USP13, then followed by pLVX-PTEN transduction. Expression of USP13 and PTEN was measured by western blot analysis. The internal control genes were GAPDH for western blot analysis, and the gels were run under the same experimental conditions. The band intensities were calculated by Image J 1.46r software, and the ratio of target gene to GAPDH was used to conduct the statistical analysis. *P < 0.05 and **P < 0.01, as determined by Student’s T-test. (PDF 4221 kb)

Additional file 3: **Figure S3**. USP13 expression was measured by western blot analysis (a) and qRT-PCR analysis (b) after USP13-shRNA transduction in 5637 cells. c. The schematic diagram of NF-kB binding site on the promoter region of mir-130b~301b cluster. d and e. Expression of NF-kB downstream targets was detected 0, 6, 12, 24 h after TNF-α treatment in 5637 and UM-UC-3 cells. f and g. expression of miR-130b-3p and miR-301b-3p was detected 0, 6, 12, 24 h after TNF-α treatment in 5637 and UM-UC-3 cells. h. NF-kB p65 was overexpressed in 5637 and UM-UC-3 cells by transfecting the pLvx-NF-kB p65 plasmids into the cells. i and j. Expression of NF-kB target genes, miR-130b-3p and miR-301b-3p was detected in BC cells transfected with pLVX-NC or pLVX-NF-kB p65. k. Western blotting analysis was performed to measure the expression of USP13 in response to Flag-USP13 plasmids transfection in 5637 cells. For real-time PCR, β-actin and U6 snRNA were used as the internal control for mRNA and miRNA, the Ct values for each group were compared using the the 2-ΔΔCt method. The internal control genes were GAPDH for western blot analysis, and the gels were run under the same experimental conditions. The band intensities were calculated by Image J 1.46r software, and the ratio of target gene to GAPDH was used to conduct the statistical analysis. *P < 0.05 and **P < 0.01, as determined by Student’s T-test. (PDF 1918 kb)

Additional file 4: **Figure S4**. The image of silver staining to visualize the binding between USP13 and PTEN protein, and the enrichment of PTEN protein after NF-kB activation. 293 T cells were transfected with Flag-tagged USP13 (lane 2 and 3) or empty vector (lane 1) for 24 h, then followed by TNF-a (lane 2) or relative vehicle treatment (lane 1 and 3) for 12 h. Lysates were subjected to immunoprecipitation with anti-Flag M2 beads. The pull-down production was then subjected to silver staining assay. In lane 3, band could be visualized at around 50 kD (PTEN), and the band at 50 kD was significant strengthened after NF-kB activation (lane 2). Bands at around 95 kD (USP13) could also be visualized in lane 2 and 3. (PDF 2701 kb)

Additional file 5: **Table S1**. qPCR primers and shRNA sequences used in the study. (XLSX 9 kb)

## Data Availability

The datasets supporting the findings of this study are included within the article.

## Abbreviations

CCL5: C-C motif chemokine ligand 5
CXCL8: C-X-C motif chemokine ligand 8
CYLD: Cylindromatosis
NF-kB: Nuclear factor kappa-B
NFKBIA: NFKB inhibitor alpha
PI3K: phosphatidylinositol 3-kinase
PTEN: phosphatase and tensin homologue deleted on chromosome 10
TNFALP3: TNF alpha induced protein 3
USP13: Ubiquitin specific peptidase 13
XIAP: X-linked inhibitor of apoptosis
