# Correction to: USP13 functions as a tumor suppressor by blocking the NF-kB-mediated PTEN downregulation in human bladder cancer

**DOI:** 10.1186/s13046-021-02194-0

**Published:** 2021-12-09

**Authors:** Xiaojun Man, Chiyuan Piao, Xuyong Lin, Chuize Kong, Xiaolu Cui, Yuanjun Jiang

**Affiliations:** 1 Department of Urology, First hospital of China Medical University, No.155 Nanjing north Road, Shenyang, 110001 Liaoning China; 2 Department of Pathology, The First Affiliated Hospital and College of Basic Medical Sciences, China Medical University, Shenyang, 110001 China

**Correction to: J Exp Clin Cancer Res 38, 259 (2019)**

**https://doi.org/10.1186/s13046-019-1262-4**

Following publication of the original article [1], the authors identified minor errors in Figs. 2, 4 and 6, specifically:

- In Fig. 2e, incorrect image was used for migration of 5637 cells after miR-301b overexpression (1^st^ row, 3^rd^ column)
- In Fig. 2h, incorrect images were used for UM-UC-3/Sh-USP13 (2^nd^ row, 1^st^ and 2^nd^ columns)
- In Fig. 4f, incorrect image was used for migration assay of UM-UC-3 (4^th^ row, 2^nd^ column)
- In Fig. 6a, incorrect images were used to demonstrate USP13 Low for both PTEN and USP13 immunohistochemistry staining (2^nd^ row, 1^st^ and 2^nd^ columns)

The corrected figures are given here. The corrections do not have any effect on the final conclusions of the paper. The original article has been corrected.

**Fig. 2:**
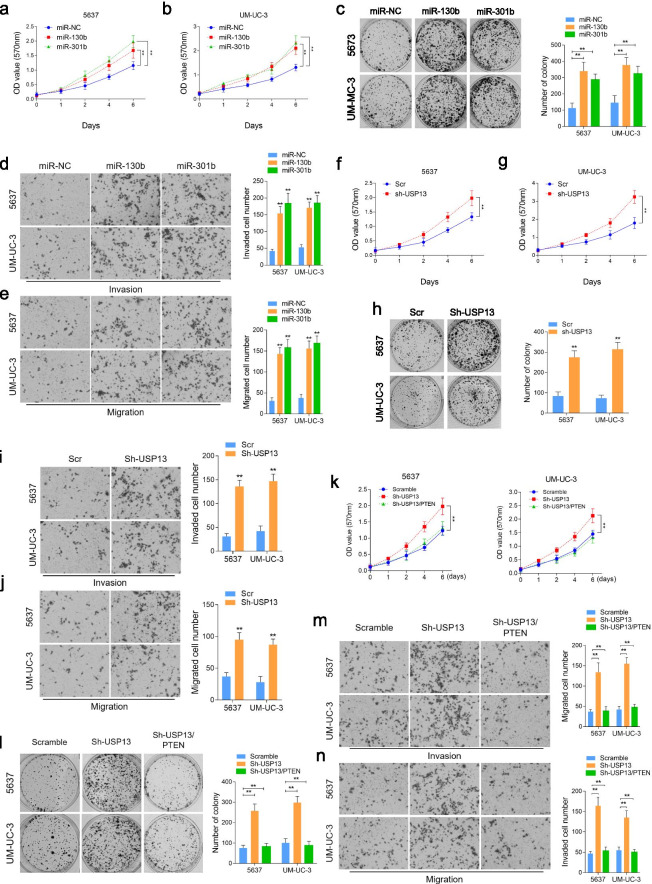
The biological function of miR-130b/301b and USP13 in vitro. Cell proliferative capacity was measured by Cell Counting-Kit 8 (CCK-8) (**a** and **b**) assay and colony formation assay (**e**) in miR-130b/301b overexpressed 5637 and UM-UC-3 cells. CCK-8 (**c** and **d**) and colony formation assay (**f**) were also performed to evaluate the cellular proliferation in USP13 knocked down 5637 and UM-UC-3 cells. Cell invasive and migrative capacities were measured by transwell assay in miR-130b/301b overexpressed (**g** and **h**) or USP13 knocked down (**i** and **j**) 5637 and UM-UC-3 cells. Cell proliferation was detected by CCK-8 (**k**) and colony formation assay (**l**) in USP13 knocked down alone or USP13 knocked down as well as PTEN expression restored 5637 and UM-UC-3 cells. Cell invasive (**m**) and migrative (**n**) capacities were measured by Transwell assay in USP13 knocked down alone or USP13 knocked down as well as PTEN expression restored 5637 and UM-UC-3 cells. Original magnification: 400×. *P < 0.05 and **P < 0.01, as determined by Student’s T-test

**Fig. 4:**
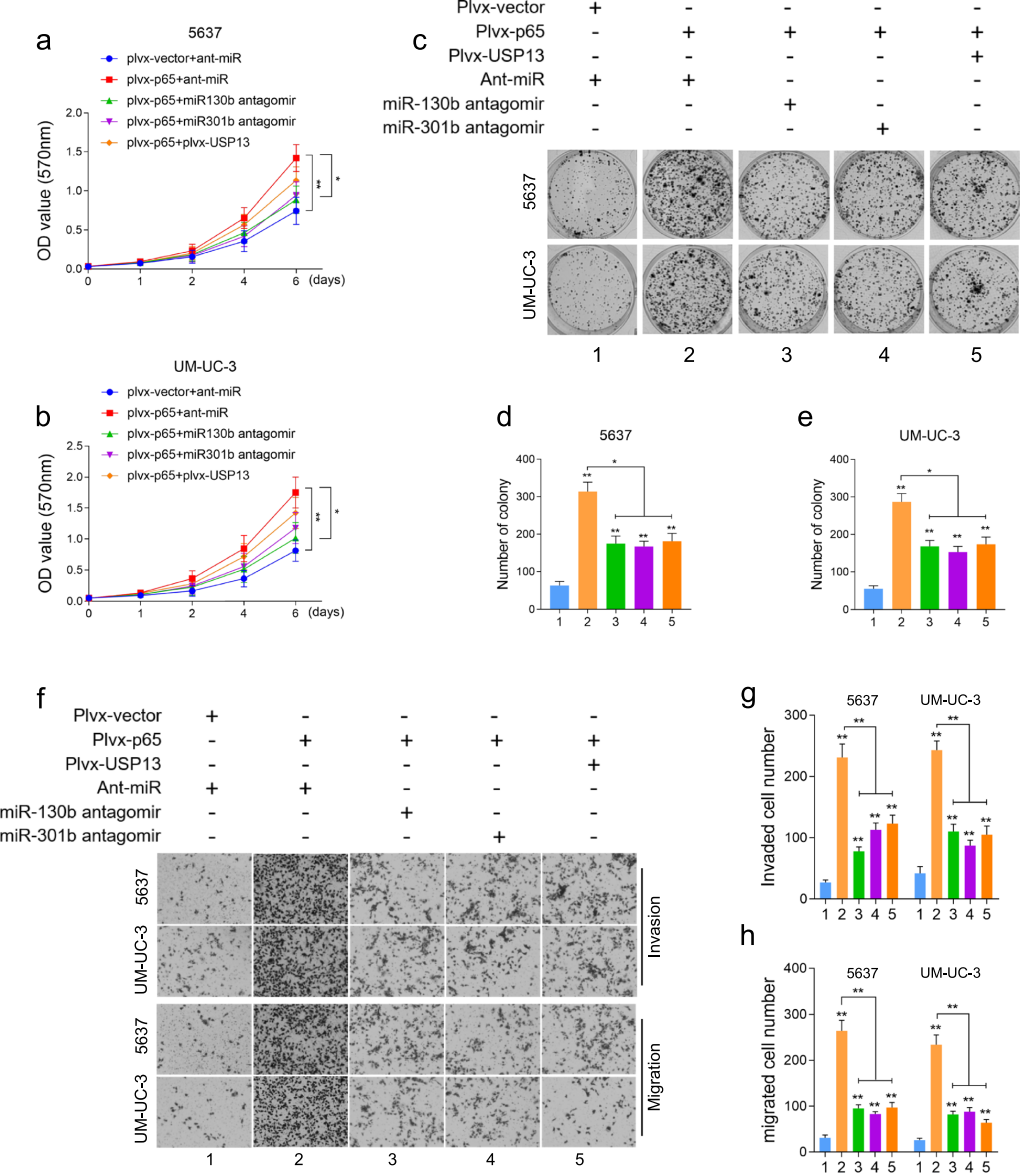
The biological function of NF-kB/miR-130b~301b/USP13 axis in vitro. **a-e**. NF-kB p65 was overexpressed in BC cells by transfecting with pLvx-NF-kB p65, then followed by transfection of antagomir of miR-130b/301b or USP13 overexpression. Cell proliferation index was determined by CCK-8 assay and cell colony formation assay. Each group was indicated as: 1 empty vector; 2 NF-kB p65 overexpression; 3 NF-kB p65 overexpression with miR-130b knockdown; 4 NF-kB p65 overexpression with miR-301b knockdown; 5 NF-kB p65 overexpression with restoration of USP13 expression. **f-h**. Cell invasive and migrative capacities were determined by Transwell assay. The cells were grouped as described in (**a**). Original magnification: 400×. *P < 0.05 and **P < 0.01, as determined by Student’s T-test

**Fig. 6:**
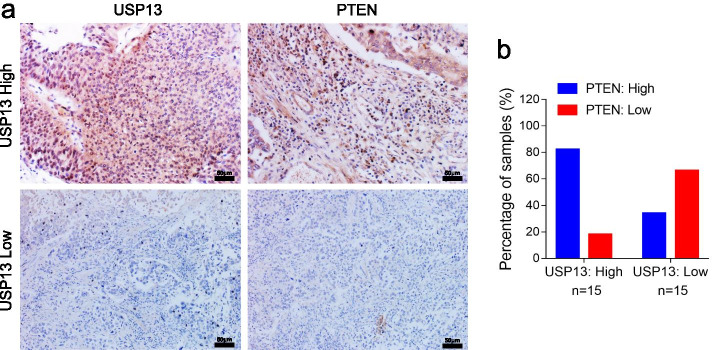
High PTEN levels correlate with USP13 overexpression in a subset of human bladder cancer tissue specimens. **a.** Representative images demonstrating PTEN and USP13 immunohistochemistry (IHC) staining of human bladder cancer tissue specimens from 30 bladder cancer patients. **b.** Quantification of PTEN or USP13 staining in BC tissue specimens. Staining intensity of PTEN or USP13 was scored as 0 to3 (0: no staining, 1: weak staining, 2: medium staining, and 3: strong staining. 0 and 1 were classified as low-expression, whereas 2 and 3 were defined as high-expression. High PTEN expression was correlated with high USP13 expression (*P* < 0.05, Fisher’s exact test)
