# Supplementary figures and images for: USP13 functions as a tumor suppressor by blocking the NF-kB-mediated PTEN downregulation in human bladder cancer
Source: J Exp Clin Cancer Res. 2019 Jun 14;38:259. doi: 10.1186/s13046-019-1262-4 (PMC6570860; doi:10.1186/s13046-019-1262-4)

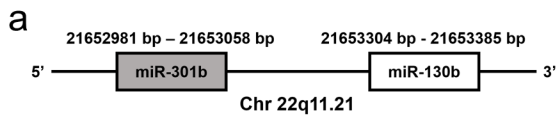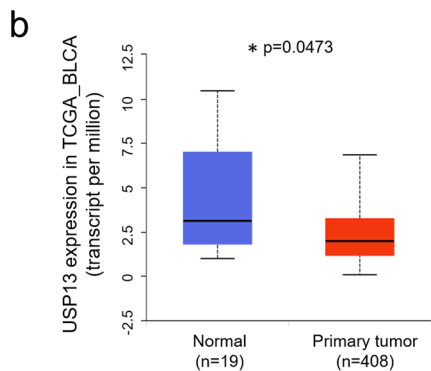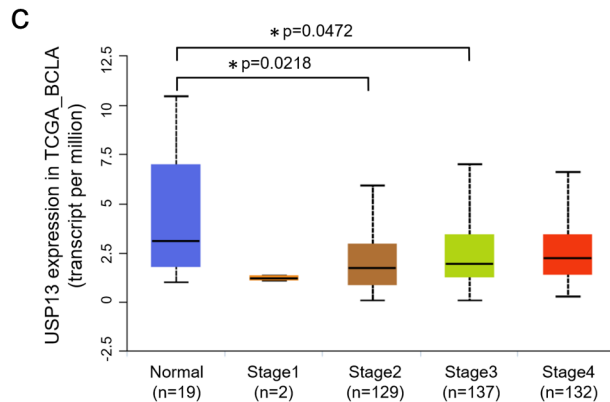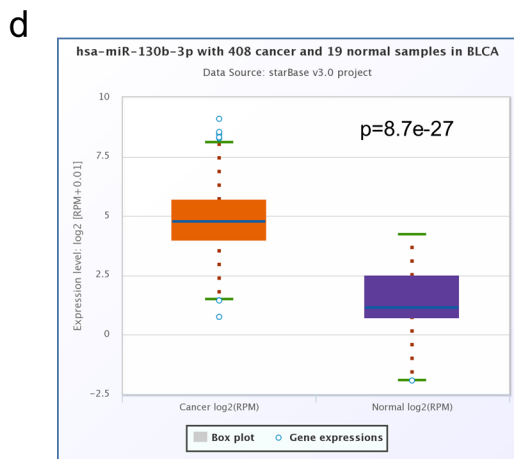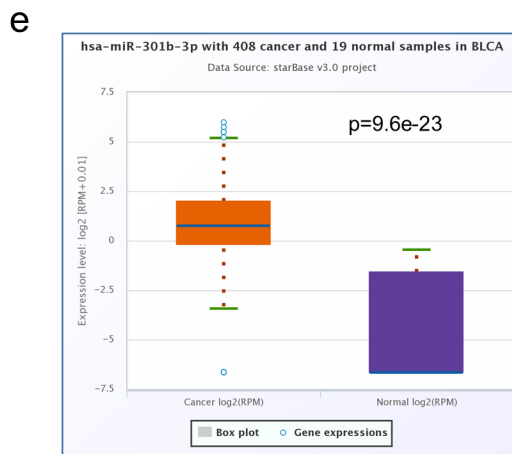

Supplement: Supplementary file 1 — Figure S1. a. The schematic diagram of mir-130b and mir-301b gene. b. Expression of USP13 in normal bladder tissues and bladder tumor tissues from TCGA dataset. c. Expression of USP13 in normal bladder tissue and bladder tumor tissues based on individual stages (TCGA). d and e. Expression of hsa-miR-130b-3p and hsa-miR-301b-3p in normal bladder tissue and bladder tumor tissue from TCGA dataset. (PDF 724 kb) [file 13046_2019_1262_MOESM1_ESM.pdf]

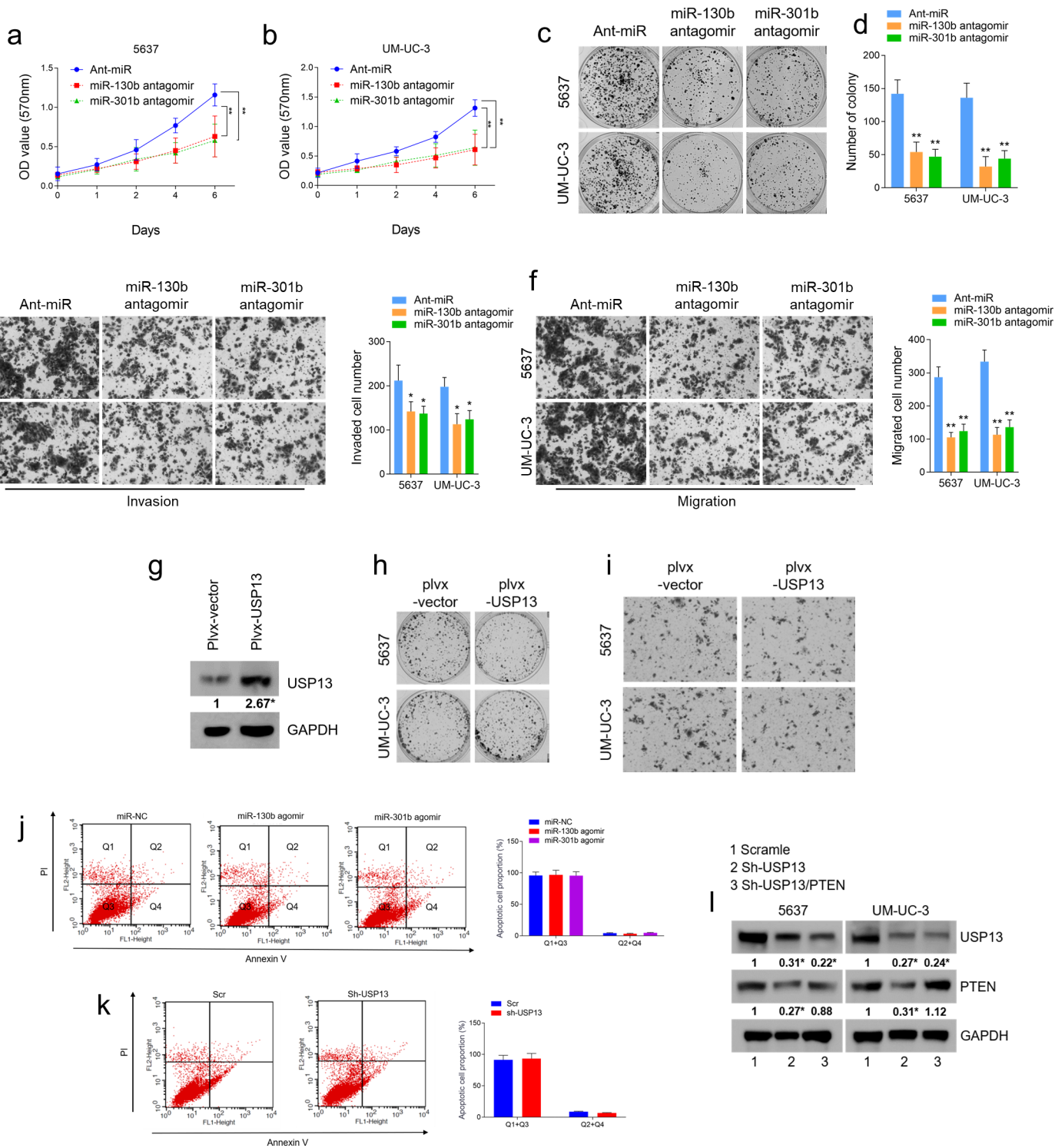

Supplement: Supplementary file 2 — Figure S2. Cell proliferation index was measured in BC cells after knockdown of miR-130b/301b by CCK-8 assay (a and b) and colony formation assay (c and d). Cell invasive and migrative capacities were measured in BC cells after knockdown of miR-130b/301b by transwell assay (e and f). Number of colonies and invaded/migrated cells was counted by Image J software. Original magnification: 400×. *P < 0.05 and **P < 0.01, as determined by Student’s T-test. g. USP13 was overexpressed by transducing the lentivirus vector encoded USP13 gene into the cells. h and i. USP13 overexpression alone doesn’t affect cell proliferation and invasion of bladder cancer cell line. j and K. no significant alterations were observed in cell apoptosis after miR-130b/301b overexpressing or USP13 knockdown in 5637 cells. i. 5637 and UM-UC-3 cells were transduced by Sh-USP13, then followed by pLVX-PTEN transduction. Expression of USP13 and PTEN was measured by western blot analysis. The internal control genes were GAPDH for western blot analysis, and the gels were run under the same experimental conditions. The band intensities were calculated by Image J 1.46r software, and the ratio of target gene to GAPDH was used to conduct the statistical analysis. *P < 0.05 and **P < 0.01, as determined by Student’s T-test. (PDF 4221 kb) [file 13046_2019_1262_MOESM2_ESM.pdf]

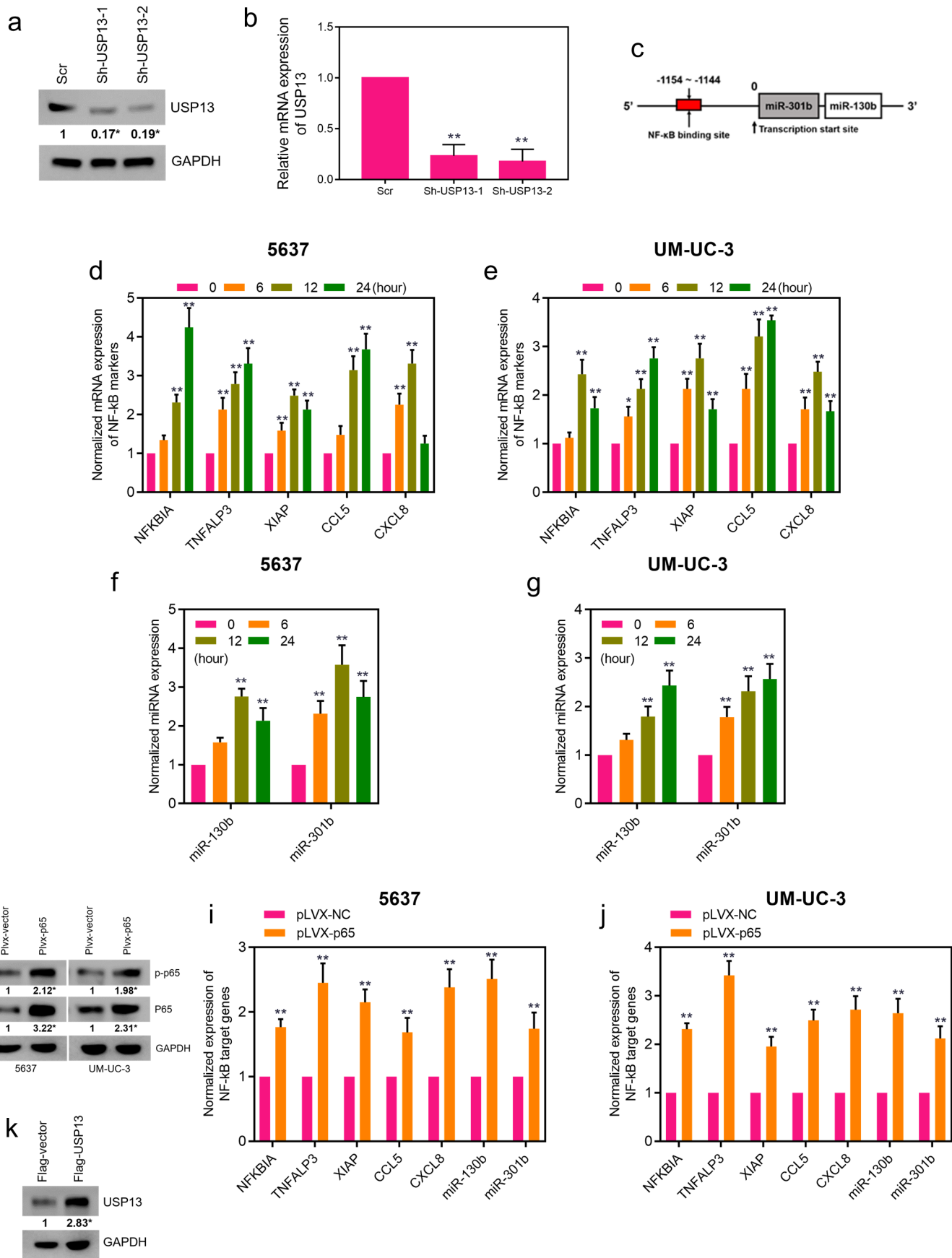

Supplement: Supplementary file 3 — Figure S3. USP13 expression was measured by western blot analysis (a) and qRT-PCR analysis (b) after USP13-shRNA transduction in 5637 cells. c. The schematic diagram of NF-kB binding site on the promoter region of mir-130b~301b cluster. d and e. Expression of NF-kB downstream targets was detected 0, 6, 12, 24 h after TNF-α treatment in 5637 and UM-UC-3 cells. f and g. expression of miR-130b-3p and miR-301b-3p was detected 0, 6, 12, 24 h after TNF-α treatment in 5637 and UM-UC-3 cells. h. NF-kB p65 was overexpressed in 5637 and UM-UC-3 cells by transfecting the pLvx-NF-kB p65 plasmids into the cells. i and j. Expression of NF-kB target genes, miR-130b-3p and miR-301b-3p was detected in BC cells transfected with pLVX-NC or pLVX-NF-kB p65. k. Western blotting analysis was performed to measure the expression of USP13 in response to Flag-USP13 plasmids transfection in 5637 cells. For real-time PCR, β-actin and U6 snRNA were used as the internal control for mRNA and miRNA, the Ct values for each group were compared using the the 2-ΔΔCt method. The internal control genes were GAPDH for western blot analysis, and the gels were run under the same experimental conditions. The band intensities were calculated by Image J 1.46r software, and the ratio of target gene to GAPDH was used to conduct the statistical analysis. *P < 0.05 and **P < 0.01, as determined by Student’s T-test. (PDF 1918 kb) [file 13046_2019_1262_MOESM3_ESM.pdf]

|               |   |   |   |
|---------------|---|---|---|
| TNF- $\alpha$ | - | + | - |
| Flag-vector   | + | - | - |
| Flag-USP13    | - | + | + |

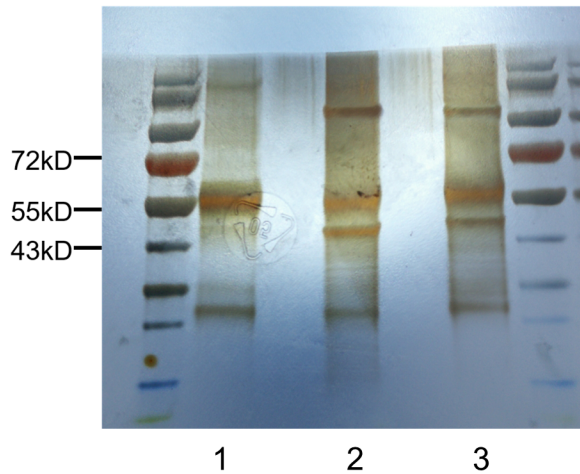

Supplement: Supplementary file 4 — Figure S4. The image of silver staining to visualize the binding between USP13 and PTEN protein, and the enrichment of PTEN protein after NF-kB activation. 293 T cells were transfected with Flag-tagged USP13 (lane 2 and 3) or empty vector (lane 1) for 24 h, then followed by TNF-a (lane 2) or relative vehicle treatment (lane 1 and 3) for 12 h. Lysates were subjected to immunoprecipitation with anti-Flag M2 beads. The pull-down production was then subjected to silver staining assay. In lane 3, band could be visualized at around 50 kD (PTEN), and the band at 50 kD was significant strengthened after NF-kB activation (lane 2). Bands at around 95 kD (USP13) could also be visualized in lane 2 and 3. (PDF 2701 kb) [file 13046_2019_1262_MOESM4_ESM.pdf]
